# Tissue Kallikrein-1 Suppresses Type I Interferon Responses and Reduces Depressive-Like Behavior in the MRL/lpr Lupus-Prone Mouse Model

**DOI:** 10.3390/ijms251810080

**Published:** 2024-09-19

**Authors:** Priyanka S. Bhoj, Cassandra Nocito, Namdev S. Togre, Malika Winfield, Cody Lubinsky, Sabeeya Khan, Nikhita Mogadala, Alecia Seliga, Ellen M. Unterwald, Yuri Persidsky, Uma Sriram

**Affiliations:** 1 Department of Pathology and Laboratory Medicine, Lewis Katz School of Medicine, Temple University, Philadelphia, PA 19140, USA; priyanka.bhoj@temple.edu (P.S.B.); cassandra.nocito@gmail.com (C.N.); namdev.togre@temple.edu (N.S.T.); malika.winfield@temple.edu (M.W.); codylubinsky@gmail.com (C.L.); sabeeyakj@gmail.com (S.K.); nikhita.mogadala@gmail.com (N.M.); aleciaseliga.as@gmail.com (A.S.); yuri.persidsky@temple.edu (Y.P.); 2 Center for Substance Abuse Research, Lewis Katz School of Medicine, Temple University, Philadelphia, PA 19140, USA; ellen.unterwald@temple.edu

**Keywords:** neuropsychiatric lupus, type I interferons, kallikrein–kinin system, tissue kallikreins, depression

## Abstract

Excessive production and response to Type I interferons (IFNs) is a hallmark of systemic lupus erythematosus (SLE). Neuropsychiatric lupus (NPSLE) is a common manifestation of human SLE, with major depression as the most common presentation. Clinical studies have demonstrated that IFNα can cause depressive symptoms. We have shown that the kallikrein–kinin system (KKS) [comprised of kallikreins (Klks) and bradykinins] and angiotensin-converting enzyme inhibitors suppressed Type I IFN responses in dendritic cells from lupus-prone mice and human peripheral blood mononuclear cells. Tissue Klk genes are decreased in patients with lupus, and giving exogenous Klk1 ameliorated kidney pathology in mice. We retro-orbitally administered mouse *klk1* gene-carrying adenovirus in the Murphy Roths Large lymphoproliferative (MRL/lpr) lupus-prone mice at early disease onset and analyzed immune responses and depressive-like behavior. Klk1 improved depressive-like behavior, suppressed interferon-responsive genes and neuroinflammation, and reduced plasma IFNα levels and proinflammatory cytokines. Klk1 also reduced IFNAR1 and JAK1 protein expression, important upstream molecules in Type I IFN signaling. Klk1 reduced bradykinin B1 receptor expression, which is known to induce proinflammatory response. Together, these findings suggest that Klk1 may be a potential therapeutic candidate to control IFNα production/responses and other inflammatory responses in SLE and NPSLE.

## 1. Introduction

Neuropsychiatric lupus (NPSLE) [1,2] is one of the common manifestations of human systemic lupus erythematosus (SLE). NPSLE symptoms include anxiety, depression, and cognitive impairment. NPSLE pathogenesis includes ischemic and neuroinflammatory mechanisms [3]. The symptoms present within the first year of lupus diagnosis [4] suggest that early factors contributing to peripheral autoimmunity may promote NPSLE.

Extensive studies in the past two decades have highlighted IFNα as a central player in lupus pathogenesis [5,6]. Elevated IFNα levels in the cerebrospinal fluid (CSF) are associated with lupus psychosis [7]. Moreover, IFNα administration in chronic viral infections and some cancers [8,9] causes depression in a high percentage of patients. Recent studies show that systemic immune responses—including Type I IFNs—affect neuroinflammation and neuropsychiatric responses [10]. Our recent publication [11] shows an increased IFN signature in the brain and increased depressive-like behavior in the Murphy Roths Large lymphoproliferative (MRL/lpr) lupus-prone mouse model at a young age compared to the age-matched wild-type strain. We hypothesize that excess IFNα (produced in the brain or coming from the periphery) causes NPSLE symptoms, and reducing this response is important to ameliorate the disease.

We discovered that the kallikrein–kinin system (KKS) can suppress the Type I IFN response [12]. The KKS [comprised of kallikreins (Klks), bradykinins (BKs)] and angiotensin-converting enzyme inhibitors (ACEi), which bridge the KKS–renin–angiotensin (RAS) pathway, suppressed the Type I IFN responses in murine dendritic cells (DCs) from normal and lupus-prone mice and in human peripheral blood mononuclear cells [12]. Tissue Klks [13], an important component of the KKS, are a family of 15 secreted serine proteases that are emerging as important regulators of neurological diseases [14,15,16], including Alzheimer’s disease [17], Parkinson’s disease [18], and multiple sclerosis [19]. Tissue Klk converts low molecular weight kininogen to kinin peptide [20], in turn activating bradykinin B1 and B2 receptors and triggering a host of biological effects [15,21,22]: blood pressure regulation, smooth muscle contraction/relaxation, vascular cell growth, vascular permeability, inflammatory cascade inactivation, and neuroprotective effects. It has been shown that ACE from the RAS pathway affects BK production and downstream signaling in cardiovascular models [15,23]; we demonstrated similar effects in our NPSLE model [11]. We hypothesize that Klks also act via signaling BK to bring about the effects in this model. They may also act via protease-activated receptors (PARs) to bring about the effects [24,25,26,27,28,29]. 

Kallikrein 1 (Klk1), a widely expressed tissue Klk [13,30], is decreased in lupus patients and lupus mouse models [31]; administration of exogenous Klk1 ameliorated lupus nephritis in mice [31,32]. Earlier studies have shown that Klk1 only affects the kidneys [31,32]. Organ-specific responses vary in lupus, and for the first time, we demonstrate the effects of Klk1 in regulating IFN responses in vivo in the periphery and in the central nervous system (CNS) (neuroinflammation and behavior) in lupus. Using the MRL/lpr mice, a popularly used lupus-prone model, we show in a short-term study that exogenous *Klk1* administration reduced depressive-like behavior, key interferon responsive genes (IRGs), and proinflammatory responses. *Klk1* administration transformed the microglia to a less inflammatory state. It also normalized the expression of key genes associated with depression.

## 2. Results

### 2.1. Adenoviral Vector Delivery of Mouse Klk1 Increases Tissue Protein Expression

**Figure 1:**
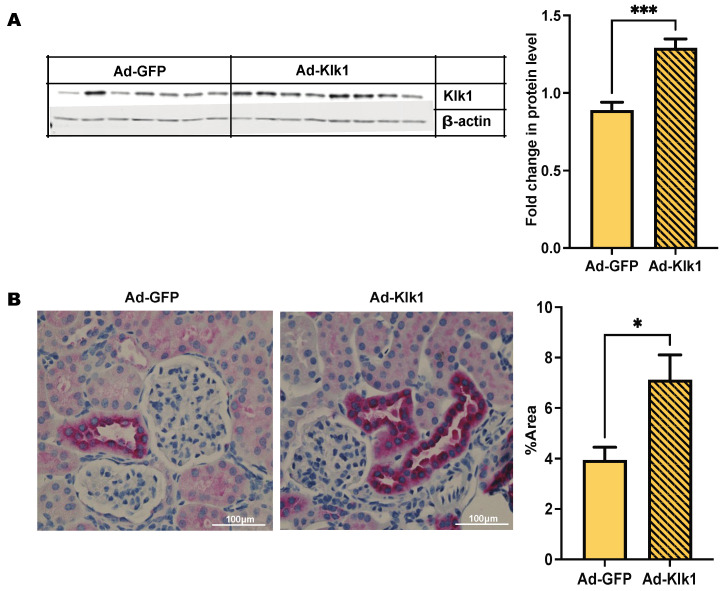
Administration of *Ad-klk1* increases Klk1 protein expression (**A**) Western blot of Klk1 protein (MW: 28 kDa) and (**B**) immunohistochemical results (indicated by pink staining) of Klk1 protein expression in the kidney of the spontaneous cohort. Images were taken at 40× magnification and analyzed using ImageJ (1.54f software). The results are expressed as mean ±SEM. An unpaired *t*-test was used; * *p* ≤ 0.05 and *** *p* ≤ 0.001 compared to the Ad-GFP group; *n =* 7–8 mice/group.

Chandra Mohan’s group has shown [32] that mouse *klk1* gene delivery using an adenoviral vector via the tail vein was able to infect and express Klk1 protein in the peripheral organs. We used a retro-orbital injection route to administer the vector and analyzed Klk1 expression by western blot and immunohistochemical analyses (Figure 1A,B). Klk1 protein expression was higher in the kidneys of the Ad-Klk1 group than in those of the Ad-GFP group.

### 2.2. Tissue Klk1 Decreases IRG and hmgb1 Gene Expression in the Brain and Periphery

**Figure 2:**
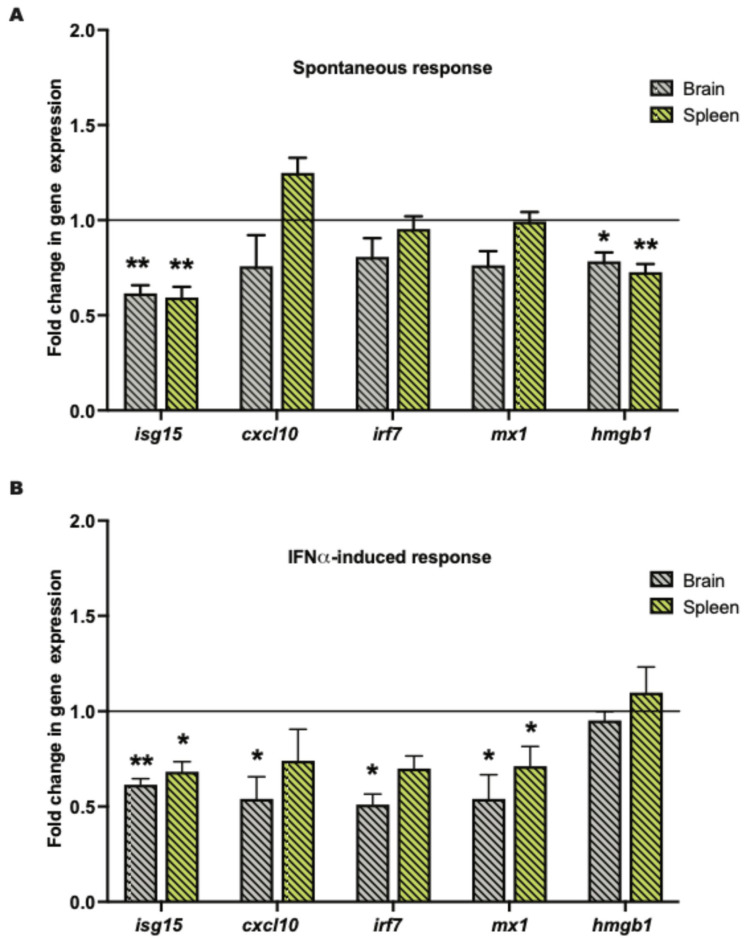
Tissue Klk1 decreases IRGs and *hmgb1* gene expression in the brain and periphery: (**A**) Spontaneous MRL/lpr mice and (**B**) IFNα-induced MRL/lpr mice. The data, obtained by qPCR, were analyzed using an unpaired *t*-test and are expressed as the mean ±SEM of the fold change in gene expression in the Ad-Klk1 group compared to the Ad-GFP group (marked as a solid black line at 1.0 on the Y axis). * *p* ≤ 0.05 and ** *p* ≤ 0.01 compared to the Ad-GFP group; *n =* 7–8 mice/group in the spontaneous cohort, and *n =* 5 mice/group in the IFNα-induced cohort.

In the spontaneous MRL/lpr cohort, *Ad-klk1* delivery significantly decreased interferon-stimulated gene 15 (*isg15*) and high-mobility group box 1 (*hmgb1*) expression in the brain and spleen compared to those in the respective tissues of Ad-GFP group. Although not significantly different, the expression of C-X-C motif chemokine ligand 10 (*cxcl10*; *p* = 0.2), myxovirus resistance gene (*mx1*; *p* = 0.06), and interferon regulatory factor 7 (*irf7*; *p* = 0.07) was also lower in the brain of the Ad-Klk1 group than in the Ad-GFP group (Figure 2A). *Ad-klk1* administration also suppressed IRGs in the IFNα-induced MRL/lpr cohort. Gene expression of *isg15* and *mx1* were significantly lower in the brain and spleen of the Ad-Klk1 group than those in the respective tissues of the Ad-GFP group. Moreover, *cxcl10* and *irf7* expression were significantly reduced in the brain of the Ad-Klk1 group, with a similar, though not significant, trend observed in the spleen (Figure 2B), suggesting profound effects of Klk1 on Type I IFN responses in the periphery and brain. Expression of *hmgb1*, a proinflammatory danger signal that is shown to be increased during the course of the disease, was also reduced significantly in the spontaneous cohort (Figure 2A) but remained unchanged in the IFNα-induced cohort (Figure 2B). Overall, Ad-Klk1 reduced the expression of IRGs and proinflammatory genes in the brain and spleen of both spontaneous and IFNα-induced MRL/lpr mice.

### 2.3. Tissue Klk1 Reduces Inflammatory Cytokine Levels in Plasma

**Figure 3:**
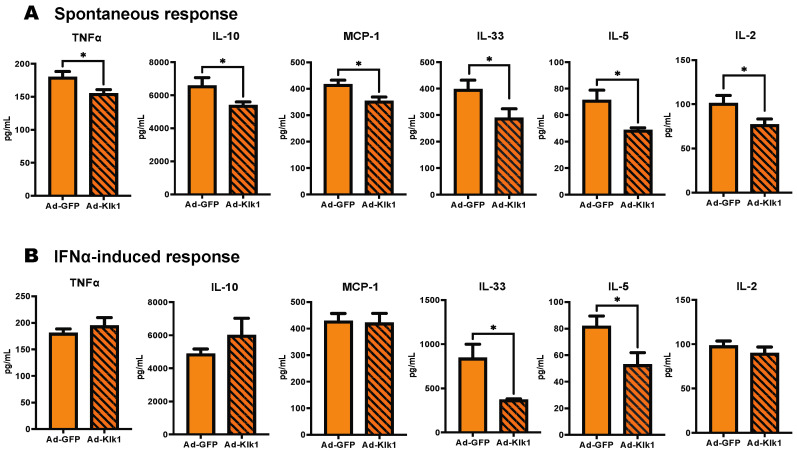
Tissue Klk1 reduces inflammatory cytokine levels in the plasma: (**A**) Spontaneous MRL/lpr mice and (**B**) IFNα-induced MRL/lpr mice. Multiplex cytokine analysis was performed using the MSD ELISA kit. The data were analyzed using an unpaired *t*-test and are expressed as the mean ±SEM. * *p* ≤ 0.05 compared to the Ad-GFP group; *n =* 7–8 mice/group in the spontaneous cohort, and *n =* 5 mice/group in the IFNα-induced cohort.

We next investigated whether Klk1 suppresses the inflammatory response in the plasma of spontaneous and IFNα-induced MRL/lpr mice. Klk1 administration significantly reduced the levels of tumor necrosis factor-α (TNFα), IL (interleukin)-10, monocyte chemoattractant protein-1 (MCP-1), IL-33, IL-5, and IL-2 in the spontaneous cohort (Figure 3A), and IL-33 and IL-5 in the IFNα-induced cohort (Figure 3B). Other proinflammatory cytokines and chemokines were unaffected or showed decreasing levels (not statistically significant) in the Ad-Klk1 group compared to Ad-GFP controls (Table S1).

### 2.4. Klk1 Decreases Plasma IFNα Levels and Proteins in Type I IFN Signaling

**Figure 4:**
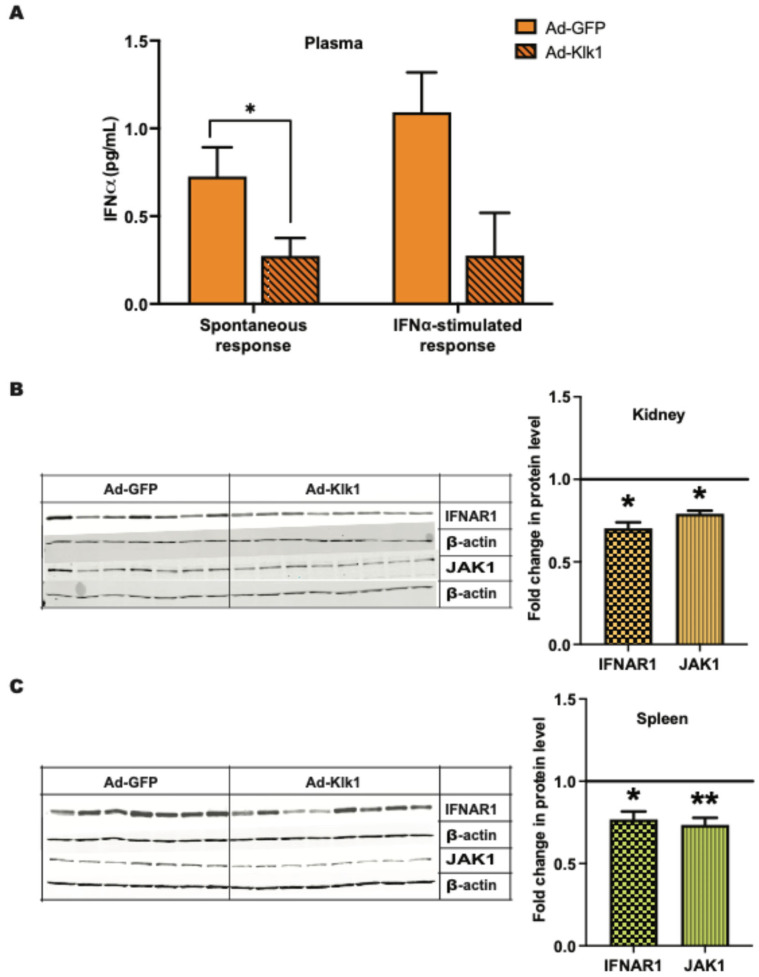
Tissue Klk1 decreases plasma IFNα levels and proteins in Type I IFN signaling: (**A**) Plasma IFNα levels in the spontaneous MRL/lpr mice were measured using the high sensitivity Verikine ELISA kit and are expressed as the mean ±SEM of IFNα levels (pg/mL). Western blot analysis of IFNAR1 (MW: 64 kDa) and JAK1 (MW: 80 kDa) protein levels in the (**B**) kidney and (**C**) spleen of spontaneous MRL/lpr mice. Data were analyzed using an unpaired *t*-test and are expressed as the mean ±SEM of the fold change in protein levels in the Ad-Klk1 group compared to the Ad-GFP group (marked as a solid black line at 1.0 on the Y axis). * *p* ≤ 0.05 and ** *p* ≤ 0.01 compared to the Ad-GFP group; *n =* 7–8 mice/group.

Increased IFNα levels in patients with SLE are a hallmark of the disease [33,34]. We tested whether Klk1 could reduce IFNα levels in the MRL/lpr mice. Circulating IFNα levels were lower in the Ad-Klk1 group than in the Ad-GFP group in both spontaneous (*p* = 0.03) and IFNα-induced (*p* = 0.06) MRL/lpr mice (Figure 4A). Type I IFN signaling follows an autocrine feedback loop via their receptor IFNAR1 that refuels and amplifies the signal [35]. We tested whether *klk1* administration can reduce IFNAR1 expression. The protein expression of IFNAR1 and JAK1, an IFNAR1-associated kinase, were significantly decreased in the kidneys (Figure 4B) and spleen (Figure 4C) following *Ad-Klk1* administration.

### 2.5. Tissue Klk1 Reduces Depressive-Like Behavior in Lupus-Prone Mice

**Figure 5:**
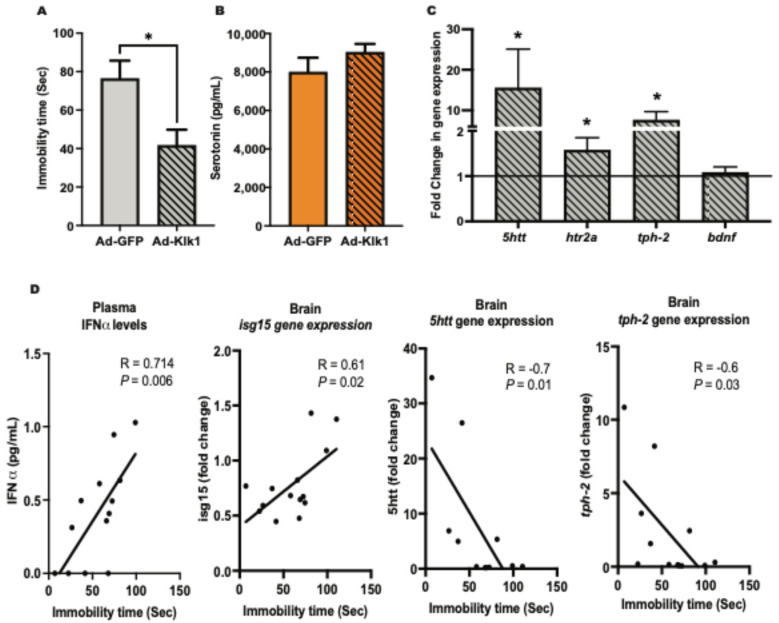
Tissue Klk1 decreases depressive-like behavior and depression-related gene expression in the spontaneous MRL/lpr mice: (**A**) Tail suspension test (TST). (**B**) Plasma serotonin levels were estimated by using the ELISA kit. (**C**) Gene expression analysis of serotonin regulators in the brain; the results are expressed as the mean ±SEM of the fold change in gene expression in the Ad-Klk1 group compared to the Ad-GFP group (marked as a solid black line at 1.0 on the Y axis). (**D**) Correlation of the immobility time in the TST with plasma IFNα levels and serotonin-related markers (brain *isg15*, *5htt*, and *tph-2*). The data were analyzed using an unpaired *t*-test and * *p* ≤ 0.05 compared to the Ad-GFP group; *n =* 7–8 mice/group.

Depression is a common psychiatric manifestation in patients with NPSLE [36]. We have previously confirmed [11] that it is an early disease manifestation in lupus-prone MRL/lpr mice [37]. Tail suspension test (TST) [38] was used to assess depressive-like behavior in our cohorts. Immobility time during the TST, a measure of depressive-like behavior, was significantly lower in the Ad-Klk1 group than in the Ad-GFP group in both spontaneous (Figure 5A) and IFNα-induced (Figure S1A) MRL/lpr mice. Notably, there was no difference in locomotor function between the groups (as measured by a rotarod test [39] for assessment of motor skills, coordination, and balance; Figure S1B), indicating that the reduced immobility time in TST is not due to a defect in locomotion but rather an indication of decreased depressive-like behavior. Although not significantly different, plasma serotonin levels, known as the body’s “feel good” neurotransmitter [40], were higher in the Ad-Klk1 group than in the Ad-GFP group (Figure 5B). The gene expression of the serotonin transporter (*5htt*), 5-hydroxytryptamine 2A receptor (*htr2a*), and tryptophan hydroxylase 2 (*tph-2*), which are the homeostatic regulators of serotonin levels in the brain, was higher in the Ad-Klk1 group than in the Ad-GFP group. However, the expression of brain-derived neurotrophic factor (*bdnf*), which also regulates depressive-like behavior, was unaltered between the groups (Figure 5C).

We further analyzed the correlation between TST immobility time and IFN-α levels, IRGs, and serotonin-related markers. Specifically, we selected markers that exhibited a significant difference between the Ad-Klk1 and Ad-GFP groups of the spontaneous cohort. Markers presented in Figure 5D showed significant correlations. TST immobility time was found to correlate positively with plasma IFN-α levels and brain *isg15* gene expression. As expected, TST immobility time was negatively correlated with the expression of the *5htt* and *tph-2* genes, both of which are key regulators of serotonin synthesis and transport.

### 2.6. Klk1 Administration Reduces Microglial Inflammatory State in the Brain

**Figure 6:**
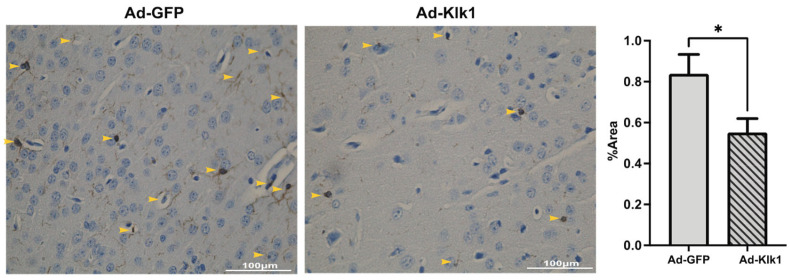
Tissue Klk1 reduced microglial inflammatory state and number. Immunohistochemical staining of IBA-1 in microglia. Data were analyzed using an unpaired *t*-test and are expressed as the mean ±SEM of the percentage area of IBA-1 staining in the cortex region of the brain at 40× magnification (brown staining, indicated by yellow arrows) using ImageJ (1.54f software). * *p* ≤ 0.05 compared to Ad-GFP group; *n =* 7–8 mice/group.

To study the effects of Klk1 on microglia in lupus-prone mice, we analyzed the expression of IBA-1, a well-established marker of microglia. We found that Klk1 transformed the microglia to a less inflammatory state (with a reduction in IBA-1 staining in Figure 6), as described elegantly by Paolicelli et al. [41].

### 2.7. Klk1 Administration Reduces C3 and IgG Expression in the Kidney

**Figure 7:**
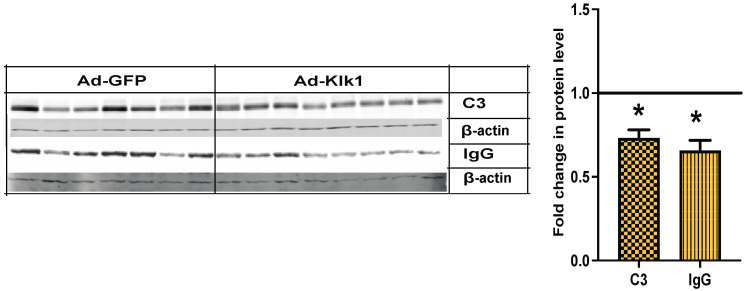
Tissue Klk1 decreases C3 and IgG protein levels in the kidney: Western blot analysis of C3 (MW: 50 kDa) and IgG (MW: 50 kDa) protein levels in the kidney of spontaneous MRL/lpr mice. Data were analyzed using an unpaired *t*-test and are expressed as the mean ±SEM of the fold change in protein levels in the Ad-Klk1 group compared to the Ad-GFP group (marked as a solid black line at 1.0 on the Y axis). * *p* ≤ 0.05 compared to the Ad-GFP group; *n =* 7–8 mice/group.

Complement and immune complex deposition in the kidney glomeruli is an early pathological hallmark of lupus nephritis [42]. Compared to *Ad-gfp* administration, *Ad-klk1* administration significantly reduced C3 and IgG protein levels in the kidneys in the spontaneous MRL/lpr cohort (Figure 7). 

We also analyzed autoantibodies against dsDNA and chromatin in the plasma in both MRL/lpr cohorts (Figure S2A,B). *Ad-klk1* administration resulted in reduced autoantibody levels, with a significant decrease in anti-dsDNA levels in the spontaneous cohort. Proteinuria and blood urea nitrogen (BUN) scores were unaltered between the groups, suggesting that a longer treatment duration may be required to observe significant changes in pathological scores.

### 2.8. Klk1 Alters BK and PAR2 Receptor Expression

**Figure 8:**
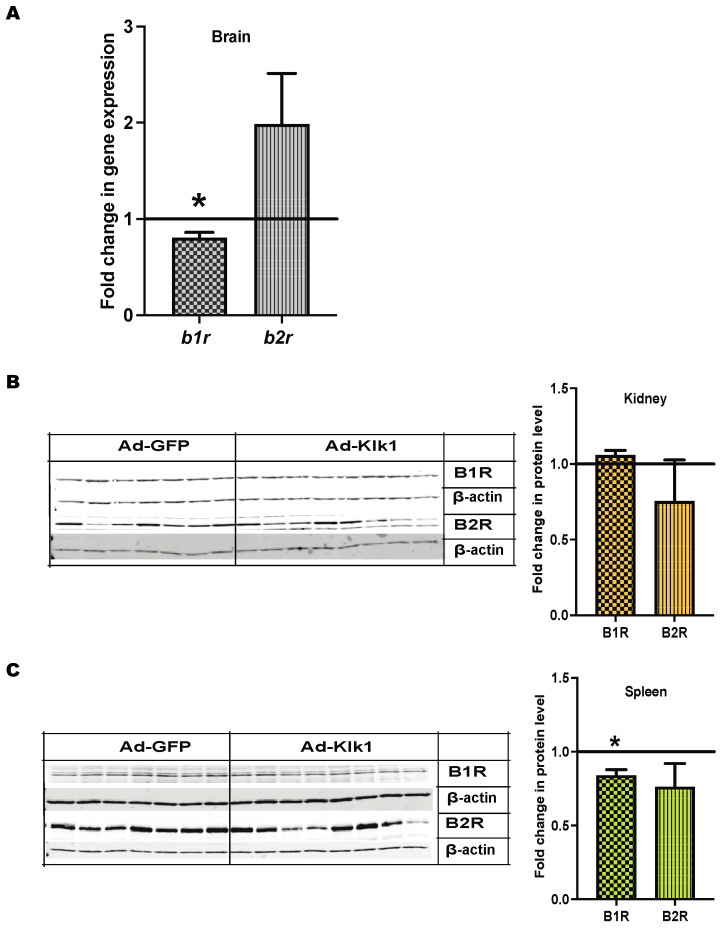
Tissue Klk1 alters BK receptor expression in the spontaneous MRL/lpr mice: (**A**) Fold change in *b1r* and *b2r* gene expression in the brain of the Ad-Klk1 group compared to those of the Ad-GFP group (marked as a solid black line at 1.0 on the Y axis), as measured by qPCR. Fold change in B1R (MW: 40 kDa) and B2R (MW: 50 kDa) protein levels in the (**B**) kidneys and (**C**) spleen of the Ad-Klk1 group compared to those of the Ad-GFP group (marked as a solid black line at 1.0 on the Y axis), as measured by western blot. The results are expressed as mean ±SEM. An unpaired *t*-test was used; * *p* ≤ 0.05 and *p* ≤ 0.001 compared to the Ad-GFP group; *n =* 7–8 mice/group.

Klk catalyzes the conversion of kininogen into BK, which activates BK receptors B1R and B2R [20]. B1R blockade has been shown to mitigate systemic autoimmunity and renal inflammation in MRL/lpr mice [43]. Therefore, we studied the effect of *Ad-klk1* administration on BK receptors’ expression. Strikingly, *b1r* gene expression was significantly lower in the brain (Figure 8A) and spleen (Figure 8C) of the Ad-Klk1 group than in the Ad-GFP group of the spontaneous MRL/lpr mice. However, no change in renal *b1r* expression was observed between the Ad-Klk1 and Ad-GFP groups (Figure 8B). Moreover, *b2r* expression was non-significantly altered in all three tissues of both groups (Figure 8); a trend in the increase in B2R gene expression was found in the brain.

Klk also signals via protease-activated receptors (PARs) [24]. Klk activation via PAR2 has been shown in various inflammatory diseases [27]. We found a significant increase in *par2* gene expression in the spleen but not in the brain and kidneys of spontaneous MRL/lpr mice after *Ad-Klk1* administration (Figure S3).

## 3. Discussion

Administration of tissue kallikrein (*klk1*) has been shown to ameliorate experimental nephritis [31,32,44]. We, for the first time, showed in this short-term study that *klk1* administration reduced Type I IFN response in the periphery and brain and normalized depressive-like behavior in the MRL/lpr mice, a popularly used NPSLE model. Klk1 also reduced autoantibody levels in the spontaneous model. The FDA recently approved anti-IFNAR treatment (anifrolumab) [45] only for severe systemic lupus treatment. Based on the rigor of previous studies in the kidney [31,32] and our data in the brain, it is evident that this molecule of the KKS could potentially be developed as a therapeutic for systemic lupus and NPSLE.

Attenuating the production and response to Type I IFNs—called the “IFN signature”—has been the goal for therapeutic development in lupus for several decades [46]. Tissue *klk1* administration could significantly suppress some of the key IRGs (IRF7 and CXCL10) both in the periphery and in the brain in this short-term analysis. In particular, *isg15* was significantly suppressed in the spontaneous and IFNα-induced cohorts. Administration of IFNα is known to induce depression in many patients with chronic viral hepatitis infection and has been an established model of inflammation-induced depression [8]. *Isg15* is one of the few genes shown to be elevated in the blood of patients with chronic viral hepatitis treated with IFNα [47]. HMGB1, released from apoptotic or necrotic cells, acts as a damage-associated molecular pattern and exerts proinflammatory effects on many cells of the innate immune system. HMGB1 has been found to play an important role in mediating inflammation and has been implicated in multiple disease phenotypes in SLE, including lupus nephritis and NPSLE [48]. In our study, Klk1 significantly reduced hmgb1 gene expression in the brain and spleen. In a recent study, a long-acting tissue Klk has been shown to reduce HMGB1 release by neurons and microglial cells after ischemic stroke [49]. 

SLE is a complex and heterogeneous disease, and NPSLE manifestations in lupus have been investigated seriously only in the last couple of decades [3]. After several clinical trials, blocking the IFNAR1 using an antagonist (anifrolumab) has only been approved recently by the FDA and only for systemic lupus [50]. Clinical trials are being revisited to understand the effects of IFNAR1 blocking, and individual case reports about the efficacy of NPSLE have been published recently [51,52]. We show in this report that *klk1* administration significantly reduced IFNAR1 as well as JAK1 signaling molecules that are upstream of Type I IFN signaling [35]. JAK inhibitors have also been independently tested in clinical trials for rheumatic diseases [53], and some are approved for rheumatoid arthritis. Our study interestingly revealed that tissue Klk1 can effectively inhibit the key signaling molecules in the Type I IFN pathway, suggesting that this is a potential candidate to develop as a biologic not only for lupus but for other IFN-mediated diseases. 

Depression and anxiety are the major psychological manifestations of NPSLE and cause severe loss of quality of life for lupus patients [3]. An estimated 40% of lupus patients suffer from the symptoms of NPSLE. Evidence from several lupus-prone mouse models has indicated IFNα as a contributor to lupus pathogenesis [10]. We have reported a constitutive increase in the IRGs of the brains of young MRL/lpr mice and an increase in depressive-like behaviors of these mice at a young age [11]. Putterman’s group has also shown [37] depression as an early and major behavioral characteristic in the MRL/lpr mice even before autoantibodies begin to develop. We have earlier reported that depressive-like behavior was the most prominent characteristic in the MRL/lpr model at an early age and sought to analyze this psychological function only in this study. Many recent studies have indicated that chronic inflammation is related to depression and mood disorders in patients [40,54]. IFNα is a well-known inducer of depression and has been studied in various models [55,56]. IFN triggers the induction of indolamine 2,3-dioxygenase (IDO) and results in higher tryptophan metabolism along the kynurenine pathway and less being available for conversion to 5-hydroxytryptophan that induces the serotonin pathway; decreased serotonin levels lead to depression. Reduced serum serotonin levels in SLE patients were related to severe disease phenotype [57,58], including nephritis, suggesting an involvement of important immunopathological processes. A Type I IFN-mediated skewing of the tryptophan metabolism and up-regulation of IDO and platelet activation were identified as possible underlying mechanisms of decreased serotonin in SLE patients [57]. While there was not a significant decrease in the circulating serotonin levels in our analysis, we observed that tissue Klk1 was able to normalize the expression of molecules in the serotonin pathway, increasing the expression of *5htt*, *htr2a*, and *tph-2*. Moreover, there was a negative correlation between immobility time and the expression of brain *5htt* and *tph-2* genes. As expected, we also found a positive correlation between circulating IFNα levels and *isg15* gene expression, which were significantly down-regulated by Klk1 administration with time of immobility in TST. BDNF, a member of the neurotrophin family, has been receiving increasing attention in SLE [59], with varied observations in patients in different studies. Studies by Zheng et al. [60] showed that decreased serum BDNF levels aggravated depression, while increased BDNF improved depression in SLE. Another report indicated that plasma BDNF levels were consistently lower in patients with NPSLE with irreversible organic brain damage than in healthy controls, and the levels increased with disease improvement. We found that in this short-term study, Klk1 increased the *bdnf* gene expression. We expect that a longer duration of treatment may lead to significant effects. 

Increased IFNα levels have been detected in the blood of SLE patients and correlate with disease activity, especially in juvenile SLE [61]. Increased IFNα has also been shown in the CSF of NPSLE patients and mouse models to be a leading effector of NPSLE. IFNα production within the CNS and increased IFNα levels in the CSF were found in patients with NPSLE. Klk1 significantly reduced circulating IFNα levels and IRGs in the brain in our short-term study. Klk1 administration also significantly reduced other key cytokines/chemokines implicated in SLE and NPSLE, such as TNFα, IL-10, and MCP-1. We have previously shown [11] a constitutive increase in proinflammatory cytokines levels, including TNFα and IL-10, in the MRL/lpr than in the MRL/wt. Effects were more pronounced in the spontaneous cohort than in the IFNα-induced cohort in this short-term study; a longer treatment period may be needed to suppress inflammation with an ongoing IFN response. Literature shows increased TNFα in patients with SLE that correlated with disease activity and has been proposed to contribute to SLE immunopathogenesis [62]. The IL-10 cytokine is specifically known for its suppressive function; however, in lupus, IL-10 is pathogenic, promoting direct differentiation of activated B cells into plasma cells [63,64]. MCP-1 has been discovered as an important biomarker for lupus nephritis activity that was high in both serums and urines of patients with lupus [65,66,67]. TNFα, IL-10, and MCP-1 (CCL2), biomarkers of inflammation, are also increased in people with depression. Interestingly, cytokines IL-33 and IL-5 were both significantly suppressed in both cohorts. We found that IL-33 was more highly induced in the IFNα-administered cohort than in the spontaneous cohort. Nonetheless, Klk1 significantly reduced the levels in both groups. IL-33, a nuclear alarmin released during cell death, elicits potent inflammatory responses. It is also implicated in lupus autoimmunity [68], and elevated serum concentrations of IL-33 and the soluble form of the IL-33 receptor, ST2L, have been reported in patients with SLE [69]. MRL/lpr lupus-prone mice treated with anti-IL-33 antibodies had reduced renal inflammation and serum autoantibodies. A recent study shows that extracellular IL-33 complexed with NETs may represent an important source of IL-33 alarmin, contributing to excessive Type I IFN [70]. Patients with lupus nephritis, an organ-specific autoimmune disease, have increased levels of IL-5 in their urine, suggesting that IL-5 may be involved in eosinophil recruitment in these patients [71]. A study reported that keratinocytes in skin lesions from patients with SLE often overexpressed IL-5 and found that higher cytokine levels correspond to more severe lesions [72]. We observed that IL-2 levels were decreased by Klk1 in the spontaneous model, while no effect was observed in the IFNα-induced cohort. IL-2 cytokine level is typically low in SLE [73]. Our observations may be an independent Klk1 response affecting T cells, probably via PARs, which needs to be investigated further. 

We have demonstrated that microglia are important players in NPSLE [74]. Microglia regulate dendrite pruning and are important for memory functions. A disease-associated microglia (DAM) population has been identified in NPSLE mouse models [75]. We have previously shown that captopril (ACEi) was able to reduce microglial activation in the MRL/lpr mice [11]. We observed that Klk1 significantly reduced the inflammatory state of microglia. We used IBA-1 as one of the markers to analyze this response, as we also showed the effects of ACE inhibitors in an earlier study [11]. As more recent studies elucidate more definitive roles of microglia [41], a further in-depth investigation is required to understand the exact role of these microglia in NPSLE. Also, whether the effects are brought about by BK that is common to both pathways remains to be investigated. 

Tissue Klk generates BK via kininogen breakdown that probably signals via the BK receptors (B1R and B2R). Des-Arg-9-BK, a breakdown product of the BK peptide (from both plasma and tissue Klk pathways) that signals via B1R, is high in rheumatic autoimmune diseases [43,76]. B1R is shown to be proinflammatory in lupus nephritis [43], and the effects can be due to stimulation with the Des-Arg-9-BK peptide. Opposing roles of kinin B1 (inflammatory) [77] and B2 receptors (protective) [78] are shown in different models. In this study, we show that Klk1 reduced B1R expression and increased B2R expression (in the brain). This can be one of the mechanisms by which Klk1 ameliorates the inflammatory effects of lupus. We showed earlier [12] in dendritic cells that blocking the BK receptors did not completely revert IFN suppression, indicating that Klk1 may act via other pathways. PARs are G protein-coupled serine protease receptors that have been shown to induce pro- and anti-inflammatory effects [24]. One of the PAR receptors, PAR2, has been reported in lupus. Recent studies [79,80] using lupus-prone mice show opposite effects via PAR2 to induce kidney disease; A PAR2 agonist has been shown to suppress IRF5 [28] (an important IFN regulatory factor implicated in lupus) in macrophages. PAR2 activation blocked apoptosis in intestinal epithelial cells [81]. We tested PAR2 expression in our cohorts and observed that *klk1* administration increased it. The elimination of inflammatory and IFN responses may be mediated via PAR2; the mechanisms need to be studied in detail. 

## 4. Materials and Methods

### 4.1. Mice

MRL/lpr female mice were purchased from the Jackson Laboratory (Bar Harbor, ME, USA) and used in experiments starting at 8 weeks (baseline). It has been shown that MRL/lpr mice start to develop autoantibodies beginning at 9 weeks of age; therefore, we started treatment in our cohort at this age. All animals were maintained in the animal facility of Temple University, an American Association for the Accreditation of Laboratory Animal Care-accredited facility, following the guidelines of the Institutional Animal Care and Use Committee of Temple University under a fully approved protocol (IACUC protocol number 5052).

### 4.2. In Vivo Gene Delivery of Ad-klk1 and Administration of IFNα

**Figure 9:**
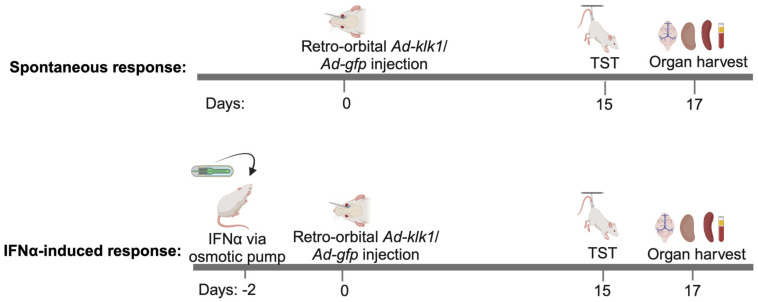
Scheme of the experiment: One cohort of nine-week-old MRL/lpr mice was administered with IFNα- or saline-carrying osmotic pump (IFNα-induced response) on day −2, followed by the retro-orbital administration of *Ad-klk1* or *Ad-gfp* vector on day 0. The spontaneous cohort received *Ad-klk1* or control (*Ad-gfp*) vector only. Behavioral studies were performed on day 15, and the mice were sacrificed on day 17 to collect the brain, spleen, kidneys, and blood.

The effects of tissue Klk1 were analyzed by using the adenovirus vector carrying the mouse *klk1* gene (*Ad-klk1*) with a GFP reporter in the spontaneous as well as IFNα-induced MRL/lpr model (Figure 9). The *Ad-klk1* was obtained from Vector Biolabs (Malvern, PA, USA) and synthesized as described previously [32]. The control vector only carried the *gfp* gene (*Ad-gfp*). 

For the spontaneous model, the mice were administered retro-orbitally (r.o.) with 1 × 10^7^ PFU (in 100 µL PBS/mouse) *Ad-klk1* or *Ad-gfp* on day 0. On days 15 and 16, a tail suspension test was performed. All mice were euthanized on day 17. For the IFNα-induced model, recombinant IFNα (5 × 10^5^ U/mouse) was administered to the mice via osmotic pumps (ALZET^®^ Osmotic Pumps, Cupertino, CA, USA) that were inserted subcutaneously following sterile procedures. Osmotic pumps were first weighed and primed with recombinant human IFNα (PBL Assay Science, Piscataway NJ, USA) or PBS for 48 h before being surgically inserted into the mice. All the pumps had a reservoir of 200 μL, which pumped 0.25 μL/h over the course of 14 days. In these experiments, *Ad-klk1* or *Ad-gfp* was administered r.o. 2 days after IFNα pump implantation. The TST was performed on day 15, and the mice were euthanized on day 17. At the terminal time point in each experiment, mice were perfused with saline, and the brain, spleen, and kidneys were harvested and stored at −80 °C for gene and protein analysis in their respective storage reagents. On day 0 and 17, blood was collected in EDTA-treated tubes for the baseline and for terminal plasma measurements, respectively. Cells were removed from the plasma by centrifugation for 10 min at 1000–2000× *g* at 4 °C.

### 4.3. Proteinuria Measurement

Proteinuria was measured using the dipstick method (Multistix 10 SG; Siemens Healthcare, Malvern, PA, USA). Briefly, mice were sacrificed using excess isoflurane. The urine was collected using a pipette and applied to dipsticks. Color was scored as per the kit instructions (a score of 1 is 30 mg/dL, 2 is 100 mg/dL, 3 is 300 mg/dL, and 4 is 2000 mg/dL) [11].

### 4.4. Blood Urea Nitrogen Analysis

After sacrificing the mice, blood was collected by sub-mandibular bleeding. BUN levels were analyzed by dipsticks (Azostix reagent strips, Siemens Healthcare, Malvern, PA, USA) and scored per manufacturer’s instructions as 1: 5–15 mg/dL, 2: 15–25 mg/dL, 3: 30–40 mg/dL, and 4: 50–80 mg/dL). Strips were scored by two personnel in a blinded fashion [11].

### 4.5. RNA Extraction and qPCR

Gene expression in the kidney, spleen, and whole-brain samples was analyzed using TaqMan probes and real-time quantitative PCR (qPCR). RNA was extracted using TriZol (Thermo Fisher Scientific, Waltham, MA, USA) following the manufacturer’s protocol. DNA removal was performed using the DNA-free^TM^ Kit (Thermo Fisher Scientific, Waltham, MA, USA) following the manufacturer’s protocol. Complementary DNA (cDNA) was synthesized with the High-Capacity cDNA Reverse Transcription Kit (Thermo Fisher Scientific, Waltham, MA, USA). 

qPCR was performed using either TaqMan primers and probes (Applied Biosystems, Foster City, CA, USA) for *klk1b11* (Mm00662365_g1), *klk1b27* (Mm00834759_gH), *irf7* (Mm00516788_m1), *isg15* (Mm01705338_s1), *cxcl10* (Mm00445235_m1), *mx1* (Mm00487796_m1), *hmgb1* (Mm00849805_gH), *b1r* (Mm04207315_s1), and *b2r* (Mm00437788_s1); or SYBR Green reagents and primers for genes listed in Table S2. *Gapdh* (Mm99999915_g1 for TaqMan reaction) was used as a reference gene. The reaction master mixes used for the TaqMan and SYBR Green systems were TaqMan™ Fast Advanced Master Mix and Applied Biosystems™ SYBR™ Green PCR Master Mix, respectively. ΔΔCt method was used to calculate the fold changes in gene expression by normalizing the values to the control group in each experiment.

### 4.6. Western Blot

Spleen and kidney tissues were homogenized and stored in the lysis buffer (Cell Lytic buffer with protease and phosphatase inhibitors added, Thermo-Fisher Scientific, Waltham, MA, USA) at −80 °C. The total protein from the samples was assayed by BCA colorimetric assay. About 30 μg of protein from each sample was loaded in a 4–20% Bis-Tris gel (Bio-Rad Laboratories, Hercules, CA, USA) after denaturation at 95 °C for 10 min. After proteins from the gel were transferred onto a nitrocellulose membrane, Ponceau staining was performed to make sure that protein transfer was even. The membrane was then blocked with a 2% blocking buffer (non-fat milk in PBS with 0.1% Tween) for 1 h and washed with PBST (PBS + 0.1% Tween). The nitrocellulose membrane was incubated overnight with the following primary anti-mouse antibodies at 4 °C in a shaking condition: anti- anti-Klk1 (Abcepta, San Diego, CA, USA, 1:1000), anti-IFNAR1 (Leinco Technologies, Fenton, MO, USA, 1:1000), anti-JAK1 (Thermo Fisher Scientific, Waltham, MA, USA, 1:1000), anti-C3 (Santacruz Biotechnology Inc, Santa Cruz, CA, USA, 1:500), anti-IgG (Abcam, Trumpington, Cambridge, UK, 1:5000), anti-B1R (Alomone Labs, Jerusalem, Israel, 1:200), anti-B2R (Alomone Labs, Jerusalem, Israel, 1:1000), and anti-β-actin (Cell Signaling, Danvers, MA, USA, 1:1000) antibodies. Membranes were washed with PBST, incubated with a secondary antibody (IR-dye-800-goat anti-rabbit antibody or IR-dye-680-goat anti-rabbit antibody, 1:10,000) for 1 h, again washed with PBST, and scanned in an Odyssey Infrared Imaging System (LI-COR Biosciences, Lincoln, NE, USA) under the 800 and 680 nm green channel for protein band visualization. ImageJ 1.54f software was used to quantify the band intensities. Band intensities were normalized to β-actin control (MW: 42 kDa).

### 4.7. Proinflammatory Cytokine Analysis

Proinflammatory cytokines and chemokine multiplex ELISA kits were bought from Mesoscale Discovery (MSD MULTI-SPOT Assay System, Rockville, MD, USA). Briefly, plasma samples and manufacturer-provided calibrator were diluted with Diluent 41 per the manufacturer’s instructions. After washing the MSD plates with 1XMSD wash buffer, 50 μL diluted samples or calibrators were added to each well and incubated for 2 h at room temperature on a shaker at 400 rpm. The plates were washed three times. A detection antibody (25 µL) was added to each well, and the plates were incubated for 2 h at room temperature on a shaker at 400 rpm. Following the incubation, the plates were washed three times, and 150 µL Read buffer T was added to each well. The plates were immediately read using an MSD instrument.

### 4.8. IFNa ELISA

IFNα levels in plasma were detected using the high-sensitivity Verikine IFNα ELISA kit from PBL (PBL Assay Science, Piscataway, NJ, USA). Briefly, standards were diluted as per the manufacturer’s instructions. Plasma samples and diluted standards were added to a plate, followed by 1 h incubation on a shaker at 400 rpm at room temperature. After washing the plate, the antibody solution was added, and the plate was incubated for 30 min at room temperature. After washing, the plate was incubated with HRP solution for 4 min at room temperature. Finally, the plate was washed and incubated with TMB substrate for 20 min in the dark, followed by the addition of the stop solution. The plate was immediately read at 450 nm. IFNα concentrations were determined by plotting the graph against standards.

### 4.9. Autoantibody Detection

Plasma was tested for anti-DNA and anti-chromatin antibodies by ELISA [82]. Briefly, polypropylene 96-well plates were coated with chicken erythrocyte-derived chromatin at 3 mg/mL or with calf thymus-derived dsDNA at 2.5 mg/mL in borate-buffered saline (BBS). Plates were coated with poly (L-lysine (1 mg/mL; Sigma-Aldrich, Allentown, PA, USA) before being coated with the antigen for anti-dsDNA ELISA. Plates were blocked with blocking buffer (3% BSA and 1% Tween 80 in BBS), and plasma samples were diluted 1:250 in BBT (0.4% Tween 80, 0.5% BSA in BBS) and added in duplicate and incubated overnight at 4 °C. Alkaline phosphatase-conjugated goat anti-mouse IgG (Fc γ specific; Jackson ImmunoResearch Laboratories, West Grove, PA, USA) was used as secondary antibody. Color was developed using 1 mg/mL para-nitrophenyl phosphate substrate (Sigma-Aldrich, Allentown, PA, USA) in 0.01 M diethanolamine (pH 9.8). Plasma from old MRL/lpr mice with high antibody titer and plasma from C57BL/6 mice were used as positive and negative controls, respectively.

### 4.10. Immunohistochemistry

Microscopic examination of the brain and kidneys was performed as per the standardized immunohistochemistry protocol [83]. Paraffin sections (5 μm) were used for the evaluation of microglia and Klk1 expression using the anti-IBA1 antibodies (Wako Chemicals, Richmond, VA, USA, 1:100) and the anti-Klk1 antibodies (Abcepta, San Diego, CA, USA, 1:100), respectively. Primary antibodies were detected by peroxidase using the Envision HRP or AP Labeled Polymer Kit (Vector Laboratories, Newark, CA, USA). Samples were imaged under 40× objective magnification using an Olympus BX31 microscope. For each mouse, four to six randomly selected fields were analyzed. ImageJ 1.54f software was used to measure the % area for each image.

### 4.11. Plasma Serotonin Analysis

Plasma serotonin levels in the plasma samples were assayed using the Serotonin ELISA Kit from Abcam (Waltham, MA, USA, cat. no. ab133053) as per the manufacturer’s instructions.

### 4.12. TST

TST was performed as a measure of depressive-like behavior after 17 days of drug administration. The total duration of immobility induced by tail suspension was measured according to the method [38]. The time during which mice remained immobile was quantified during a test period of 6 min. Mice were considered immobile only when they hung passively and were completely motionless. The immobility was scored by two independent readers and were blinded for analysis.

### 4.13. Rotarod Test

The rotarod test was used in the mice to check for locomotor function. The mice were placed on a horizontally oriented, rotating cylinder (rod) suspended above a cage floor. The rotating cylinder was high enough to induce avoidance of falls but not high enough to injure the animal. Three trials were performed, with the latency and speed at fall recorded under the following parameters: duration of trial = 5 min, maximal speed = 20 RPM, and time to maximal speed = 15 s [84].

### 4.14. Statistical Analysis

Prism 10.3.1 software (GraphPad, San Diego, CA, USA) was used for statistical analysis. An unpaired *t*-test was used for comparison between the two groups, and the results are shown as mean ±SEM. Pearson correlation analysis was applied to measure the correlations between immobility time (Sec) and IFNα, IRGs, or serotonin-related markers. *p* < 0.05 were considered significant: * *p* ≤ 0.05, ** *p* ≤ 0.01, *** *p* ≤ 0.001.

## 5. Conclusions

**Figure 10:**
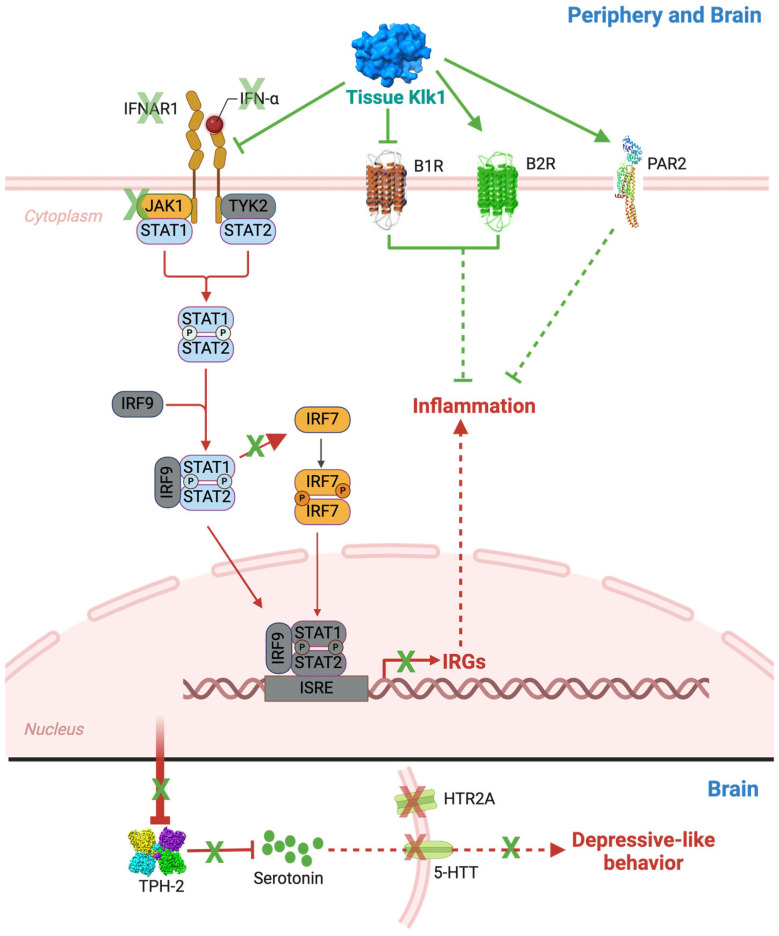
Tissue Klk1 suppresses Type I IFN responses through IFNAR1, BRs, and PAR2 and reduces depressive-like behavior in the MRL/lpr lupus-prone mice: The present study demonstrates that tissue Klk1 administration reduces IFNα, IFNAR1, JAK1, and IRF7, consequently suppressing IRG expression. Our previous study [11] has shown a reduction in signal transducer and activator of transcription (STAT) phosphorylation (shown in blue color) following bradykinin (BK) administration, which, along with other pathway proteins (shown in grey color), will be studied in detail in the future. We observed a consequential reduction in the levels of tryptophan hydroxylase 2 (TPH2, a rate-limiting enzyme in serotonin biosynthesis), serotonin (5-HT), serotonin transporter (5-HTT), and 5-hydroxytryptamine receptor 2A (HTR2A, a serotonin receptor). We also observed depressive-like behaviors in MRL/lpr mice. Alleviation of inflammatory and IFN responses may be mediated via the alteration in BK receptors (B1R and B2R) and protease-activated receptor-2 (PAR2) expression; the mechanisms need to be studied in detail (indicated with dashed arrows). The therapeutic effects of Klk1 are shown in green notation, and SLE and NPSLE pathogenesis are represented in red notation.

In all, our study shows that tissue Klk1 has the potential to ameliorate systemic as well as CNS lupus by reducing Type I IFN production and inflammatory response in the periphery and brain (Figure 10). The consequential depressive-like behavior response is also reduced. Our previous report indicated this reduction may be mediated by partially signaling via the B1/B2 receptors or PARs. In this study, we have shown that Klk1 reduced the proinflammatory B1R expression and increased B2R and PAR2 expression, which may also be protective. The mechanistic aspects of Klk1 action need to be investigated further.

## Data Availability

The data that support the findings of this study are available from the corresponding author upon reasonable request.

## Supplementary Materials

The following supporting information can be downloaded at https://www.mdpi.com/article/10.3390/ijms251810080/s1.
