# Supplementary material for: Tissue Kallikrein-1 Suppresses Type I Interferon Responses and Reduces Depressive-Like Behavior in the MRL/lpr Lupus-Prone Mouse Model
Source: Int J Mol Sci. 2024 Sep 19;25(18):10080. doi: 10.3390/ijms251810080 (PMC11432477; doi:10.3390/ijms251810080)
Supplement: Supplementary file 1 [file ijms-25-10080-s001.zip › ijms-3163507-supplementary.pdf]

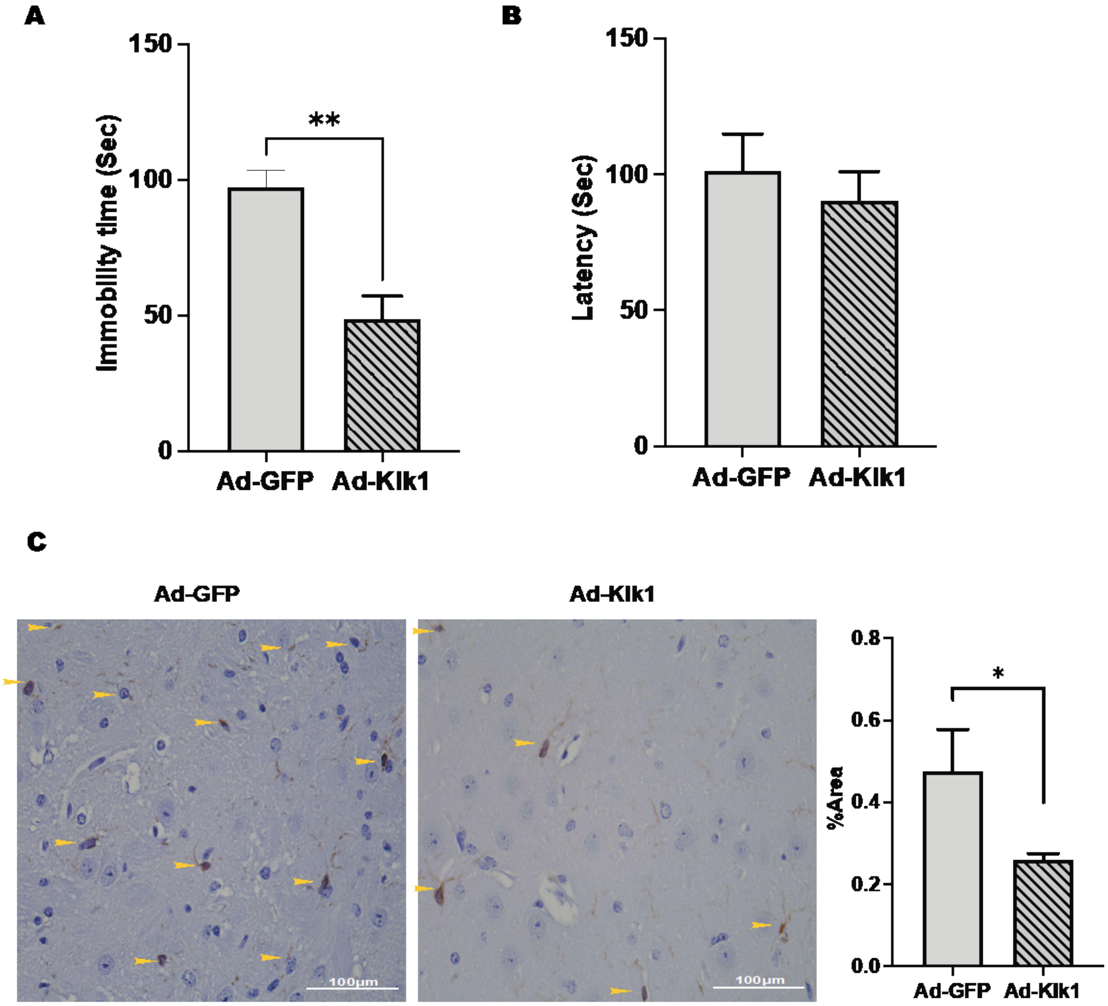

Figure S1. Tissue Klk1 decreases depressive-like behavior and microglial number in the IFNα-induced MRL/lpr mice: (A) Tail suspension test (TST) findings show significantly lower immobility time in the Ad-Klk1 group than in the Ad-GFP group. (B) Rotarod test results indicate no difference in locomotor function, suggesting that the reduced immobility time in TST is due to decreased depressive-like behavior rather than a locomotor defect. (C) Immunohistochemical staining of IBA-1 in microglia reveals a decreased number of activated microglia following *Ad-klk1* treatment. Data were analyzed using an unpaired t-test and are expressed as the mean ± SEM of the percentage area of IBA-1 staining at 40× magnification (brown staining, indicated by yellow arrows) using ImageJ software. **P*≤0.05 compared to Ad-GFP group; n = 5 mice/group.

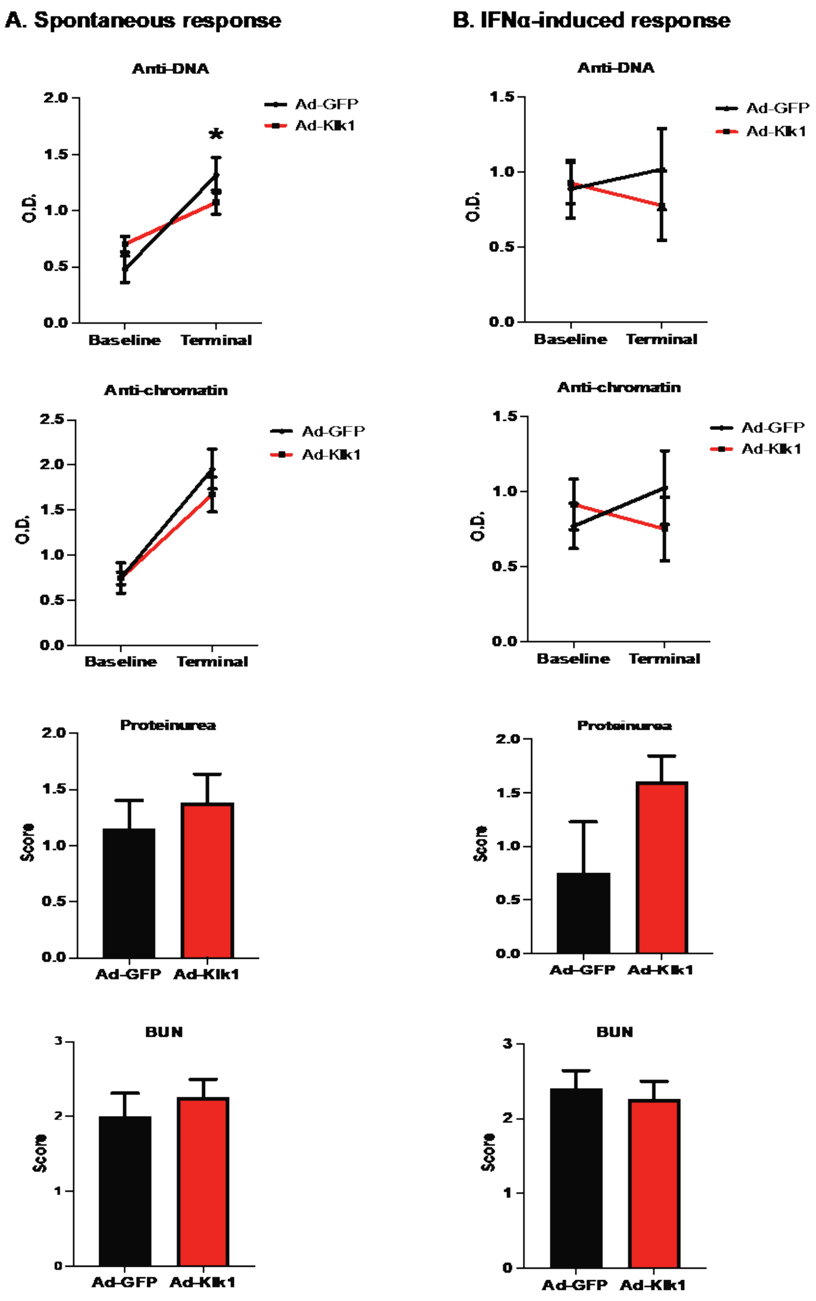

Figure S2. Tissue Klk1 decreases auto-antibody levels in the plasma of the MRL/lpr mice: (A) Spontaneous cohort. (B) IFNα-induced cohort. *Ad-klk1* administration reduced plasma autoantibody levels against dsDNA and chromatin, with a significant decrease in anti-dsDNA antibody levels in the spontaneous cohort. Proteinuria and blood urea nitrogen (BUN) scores were unaltered between the groups. Data were analyzed using an unpaired t-test and are expressed as the mean ± SEM. **P*≤0.05 compared to Ad-GFP group; n = 5–8 mice/group.

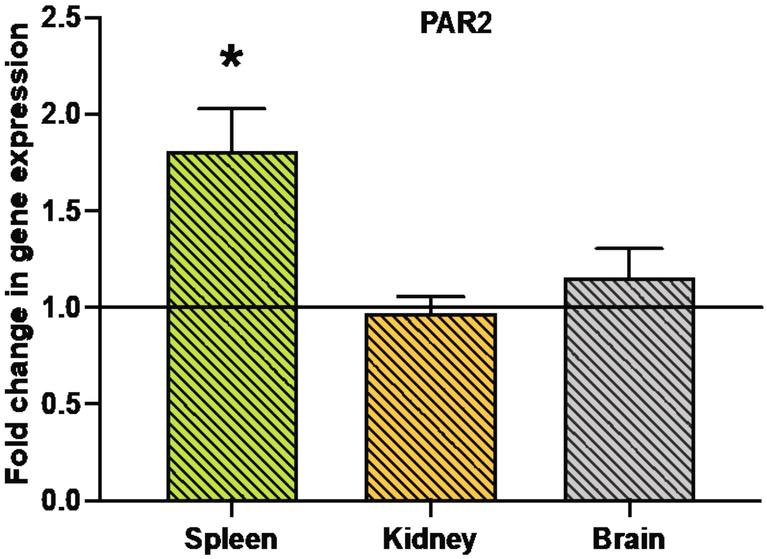

Figure S3. Tissue Klk1 increases *par2* gene expression in the spontaneous MRL/lpr mice: Ad-klk1 administration increases *par2* gene expression in the spleen but no significant change in the brain and kidneys. The data, obtained by qPCR, were analyzed using an unpaired t-test and are expressed as the mean ± SEM of the fold change in gene expression in the Ad-Klk1 group compared to the Ad-GFP group (marked as a solid black line at 1 on the Y axis). *$P \leq 0.05$ and **$P \leq 0.01$ compared to Ad-GFP group; n = 7–8 mice/group.

Table S1: Tissue Klk1 decreases proinflammatory cytokines in the plasma

	Spontaneous response			IFNα-induced response		
	**Ad-GFP**	**Ad-Klk1**	**P value**	**Ad-GFP**	**Ad-Klk1**	**P value**
**IFNγ**	4.0±0.6	3.8±0.2	0.807	3.4±0.6	4.1±1.1	0.61
**IL-4**	10.2±5.1	6.8±4.5	0.860	4.3±4.3	10.32±6.3	0.4
**IL-9**	10.23±10.2	3.41±3.4	0.51	72.6±66	0	0.3
**IL-1β**	51.8±10.71	40.6±2.7	0.3	67.75±7.1	59.41±5.3	0.37
**IL-17A**	77.42±4.2	75.22±5.7	0.76	57.4±14.4	49.3±15.4	0.74
**MIP-1a**	93.8±4.7	89.0±5.9	0.55	95.24±7.7	86.16±5.7	0.37
**IL12P70**	107.2±77.6	125.7±125.7	0.9	0	95.4±95.4	0.34
**MIP-2**	350±36	342±24	0.8633	319.4±59.6	262.9±21.3	0.3
**IL-6**	372.5±73.5	402.6±73.8	0.77	529±135.8	286±32.2	0.11
**KC/GRO**	604.2±37.2	663.2±39.7	0.3	666.5±140	577.6±67.4	0.58
**IL-15**	767.4±387.3	1260±265.8	0.3	1290±380.1	766.2±420.9	0.38
**IL-27 P28/IL-30**	863.9±46.5	819.2±42.1	0.48	759.4±85.3	806.8±95.2	0.72
**CXCL10**	946.5±55.3	852.1±28.1	0.13	705.6±40.6	776±41.6	0.26

Results are shown as mean of pg/mL of cytokine or chemokines ± SEM.

Table S2: List of primers used for qPCR by SYBR Green method

Genes studied	Forward primer 5'—3'	Reverse primer 5'—3'
5htt	TATCCAATGGGTACTCCGCAG	CCGTTCCCCTTGGTGAATCT
htr2a	TAATGCAATTAGGTGACGACTCG	GCAGGAGAGGTTGGTTCTGTTT
tph2	GGTTGTCCTTGGATTCTGCTG	GCCTGGATTCGATATGAAGCAT
bdnf	TCATACTTCGGTTGCATGAAGG	AGACCTCTCGAACCTGCCC
par2	CCGGACCGAGAACCTTG	CGGAAGAAAGACAGTGGTCAG
gapdh	AGGTCGGTGTGAACGGATTTG	TGTAGACCATGTAGTTGAGGTCA
